# Soybean F-Box-Like Protein GmFBL144 Interacts With Small Heat Shock Protein and Negatively Regulates Plant Drought Stress Tolerance

**DOI:** 10.3389/fpls.2022.823529

**Published:** 2022-06-02

**Authors:** Keheng Xu, Yu Zhao, Yan Zhao, Chen Feng, Yinhe Zhang, Fawei Wang, Xiaowei Li, Hongtao Gao, Weican Liu, Yan Jing, Rachit K. Saxena, Xianzhong Feng, Yonggang Zhou, Haiyan Li

**Affiliations:** ^1^College of Life Sciences, Jilin Agricultural University, Changchun, China; ^2^College of Tropical Crops, Sanya Nanfan Research Institute, Hainan University, Haikou, China; ^3^Hainan Yazhou Bay Seed Laboratory, Sanya, China; ^4^International Crops Research Institute for the Semi-Arid Tropics (ICRISAT), Hyderabad, India; ^5^Key Laboratory of Soybean Molecular Design Breeding, Northeast Institute of Geography and Agroecology, Chinese Academy of Sciences, Changchun, China

**Keywords:** soybean, F-box protein, drought stress, Skp1-Cullin1-F-box (SCF) complex, segmental duplication, protein interaction

## Abstract

The *F-box* gene family is one of the largest gene families in plants. These genes regulate plant growth and development, as well as biotic and abiotic stress responses, and they have been extensively researched. Drought stress is one of the major factors limiting the yield and quality of soybean. In this study, bioinformatics analysis of the soybean *F-box* gene family was performed, and the role of soybean *F-box-like* gene *GmFBL144* in drought stress adaptation was characterized. We identified 507 *F-box* genes in the soybean genome database, which were classified into 11 subfamilies. The expression profiles showed that *GmFBL144* was highly expressed in plant roots. Overexpression of *GmFBL144* increased the sensitivity of transgenic *Arabidopsis* to drought stress. Under drought stress, the hydrogen peroxide (H_2_O_2_) and malonaldehyde (MDA) contents of transgenic *Arabidopsis* were higher than those of the wild type (WT) and empty vector control, and the chlorophyll content was lower than that of the control. Y2H and bimolecular fluorescence complementation (BiFC) assays showed that GmFBL144 can interact with GmsHSP. Furthermore, our results showed that GmFBL144 can form SCF*^FBL144^* (E3 ubiquitin ligase) with GmSkp1 and GmCullin1. Altogether, these results indicate that the soybean F-box-like protein GmFBL144 may negatively regulate plant drought stress tolerance by interacting with sHSP. These findings provide a basis for molecular genetics and breeding of soybean.

## Introduction

Abiotic stress is the primary factor limiting plant growth and crop yield. Abiotic stresses include drought, saline-alkali, high/low temperature, and metal stress, of which drought stress is the most common stress (Baldoni et al., 2015; Gong et al., 2020). Plants have evolved multiple strategies to deal with drought stress. Common strategies include reducing water loss, maintaining chlorophyll content, and reducing reactive oxygen (Ritchie et al., 1990; Zafari et al., 2020). Molecular breeding has long been expected to improve crop drought tolerance. Thus far, many genes involved in drought stress regulation have been identified. For instance, *ZmVPP1*, a vacuolar-type H^+^ pyrophosphatase gene, can improve the drought tolerance of transgenic maize by enhancing root development and photosynthetic efficiency (Wang X. et al., 2016). Tubby-like F-box protein 8 (SlTLTP8) enhances plant drought tolerance by reducing water loss to improve water-use efficiency (Li et al., 2020b). Additionally to the above positive regulatory genes, some negative regulatory genes have been found. For example, MdSE reduces the expression of *MdNCED3* by negatively regulating the MdMYB88 and MdMYB124 transcription factors (MdMYB88, MdMYB124, *MdNCED3*; positive regulators of drought resistance) to reduce the drought tolerance of apples (Li et al., 2020c). Although many genes responding drought stress have been investigated, the molecular network of plant responses to drought stress is still imperfection.

The ubiquitin-proteasome system (UPS) and molecular chaperone system play important roles in plant responses to drought stress. Protein degradation mediated by UPS is an important post-translation regulation mechanism, which includes ubiquitin, ubiquitin-activating enzymes (E1s), ubiquitin conjugating enzymes (E2s), ubiquitin ligase enzymes (E3s), and 26S proteasomes (Sadanandom et al., 2012; Xia et al., 2020; Ban and Estelle, 2021). Ubiquitin is activated by E1 in the presence of ATP, and is then transferred to the cysteine residue of E2. Then, E3 transfers ubiquitin to the lysine residue of the substrate, and finally 26S proteasome degrades the ubiquitinated substrate. In this pathway, E3s are responsible for substrate recognition and substrate ubiquitination. Research has found that there are thousands of E3s in plants, which can be classified as many different types, of which RING E3s are the most abundant (Zheng and Shabek, 2013; Morreale and Walden, 2016). SCF is a well characterized RING E3 ubiquitin ligase, which contains four subunits: Cullin1 (CUL1), Ring-box protein (Rbx), S-Phase kinase associated protein 1 (Skp1), and F-box protein. Cullin1, Rbx, and Skp1 interact to form a core scaffold with ligase activity, and the F-box proteins are responsible for substrate recognition (Zheng et al., 2016; Zhang S. et al., 2019).

Recently, large numbers of F-box proteins were identified. Especially in plants, there are hundreds of F-box proteins. Their common feature is that the N-terminal contains a relatively conservative F-box domain that interacts with Skp1 and CUL1 to form SCF. Besides the F-box domain, there are other variable domains in the C-terminal. The C-terminal domains are responsible for substrate recognition and also provide a foundation for subfamily classification of F-box proteins. The diversity of F-box proteins can help SCF distinguish and recruit multiple substrates. Therefore, it is not surprising that F-box proteins can regulate many physiological processes. For example, Carbonnel et al. (2020) found that F-box protein MAX2 can inhibit the growth of primary roots and promote the growth of root hairs by increasing the content of ethylene through the karrikins signaling pathway. Another case in point is that ORE9, an F-box protein, can regulate leaf senescence through the ubiquitin-proteasome pathway (Woo et al., 2001; Zhang et al., 2016). In addition, in recent years, a growing number of F-box proteins involved in abiotic stress responses have been studied, e.g., drought, salt, ion, and low temperature stresses (Lim et al., 2019; Zhang Y. et al., 2019; Jie et al., 2020; Venkatesh et al., 2020).

Presently, most research on F-box protein function are from other plants, and research on soybean F-box proteins is relatively limited. Soybean is an important food crop and oil crop, and its yield is seriously affected by drought stress. Therefore, it is necessary to study the function of GmF-box proteins in drought. In this study, we performed functional characterization of *GmFBL144* in drought stress adaptation. The results showed that overexpression of *GmFBL144* significantly reduced plant drought tolerance. Under drought stress, the overexpression lines had higher hydrogen peroxide (H_2_O_2_) and malonaldehyde (MDA) content, lower chlorophyll content, and a higher water loss rate compared to the controls [wild type (WT) and empty vector]. Furthermore, our results showed that GmFBL144 can form SCF*^FBL144^* (E3 ubiquitin ligase) with GmSkp1 and GmCulinl1, and GmFBL144 can interact with GmsHSP.

## Materials and Methods

### Identification and Bioinformatics Analysis of *GmF-Box* Genes

The whole genome protein sequence of soybean (Glycine max Wm82.a2.v1) was downloaded from the plant genome database^1^. Simultaneously, the hidden Markov model of F-box domain (PF00646) and F-box-like domain (PF12937) were downloaded from the Pfam database^2^. The proteins sequences were searched by conserved domain using the BLAST method in the protein database. The genes of the two families were merged and then identified using the NCBI database (*E*-value cutoff 1.0). The genes without the F-box domain and F-box-like domain were deleted.

Phylogenetic analysis was inferred using the Maximum Likelihood method based on the Poisson correction model by MEGA7 (1000 bootstrap replicates for detection reliability). The annotation file GFF3 for soybean was downloaded from the Phytozome database, and visualized using TBtools. Gene duplication analyses were performed with the MCScanX (Wang et al., 2013).

A 2,000 bp DNA sequence upstream of *GmFBL144* gene was download from the soybean genome database, and the promoter elements were analyzed using the PlantCARE website^3^ (Lescot et al., 2002).

### Plant Materials and Growth Conditions

The soybean genotype Williams 82 was cultivated in the field. Roots, steams, leaves, flowers, and embryos of different stages were collected and stored at −80°C for RNA extraction for quantitative RT-PCR assay. *Arabidopsis thaliana* were cultivated in a controlled-climate room with 16/8 h light/dark cycle at 23°C and 60% relative humidity.

### Generation of Transgenic *Arabidopsis*

The full-length coding sequence (CDS) of *GmGBL144* was inserted after the CaMV35S promoter, resulting in overexpression of recombinant vectors. After sequencing, the fusion constructs were transformed into Agrobacterium tumefaciens (GV3101) and then transformed into *Arabidopsis* (Col-0) plants using the floral dip (Clough and Bent, 2010). T1 seedlings were screened with 1% glufosinate and then re-confirmed using genomic PCR. After screening of the separation ratio and high expression, three homozygous T3 transgenic lines (L16, L17, and L19) were selected for phenotypic experiments.

### Stress Treatments

For rapid assay of the function of *GmFBL144* in the drought response, the full-length CDS of *GmGBL144* was inserted in pYES2 vector. The fusion construct was transformed into INVSc1 yeast competent cells using the PEG/LiAc-mediated method and was selected for uracil prototrophy. The function assay was implemented on YPD plate with 1.0 M mannitol. The cultures were grown for 3–7 days at 30°C.

After harvesting transgenic *Arabidopsis* seeds, we planted the seeds on 1/2 MS medium. These seedings were transferred to plate of 1/2 MS with 250 mM mannitol. The root length and fresh weight were measured 2 weeks later. To further confirm the function of *GmFBL144*, the transgenic *Arabidopsis* were subjected to drought (no irrigation for up to 10 and 20 days) during the rosette stage. Rehydration was implemented after 20 days of drought. *Nicotiana benthamiana* were cultivated in a controlled-climate room with 14/10 h light/dark cycle at 26°C and 50% relative humidity.

### Quantitative Real-Time PCR Analysis

Total RNA was extracted from the different soybean tissues using RNAiso Plus (Takara, DaLian, China). The cDNA was synthesized using the PrimeScript™ RT reagent kit (Takara). Quantitative real-time PCR (qRT-PCR) was performed using TB Green Premix Ex Taq™ II (Takara). The relative expression was calculated using 2^–Δ^ *^Ct^* (Li et al., 2021). The primers of qRT-PCR are listed in Supplementary Table 1.

### Subcellular Localization of GmFBL144 Proteins

The full-length *GmFBL144* gene without stop codon was inserted after the CaMV35S promoter to generate a 35S:GmFBL144-GFP fusion construct. The fusion construct was transformed into *Agrobacterium tumefaciens* (GV3101, pSoup-p19) and then transferred into epidermal *Nicotiana benthamiana* cells for transient assays. The fluorescent signals of green fluorescent protein (GFP) were observed using a laser confocal microscope (TCS SP8, Leica, Germany).

### Protein Interaction Assay

The prey cDNA library was constructed using CloneMiner II cDNA Library Construction Kit (Invitrogen: A11180) for screening interacting proteins. The full-length *GmFBL144* gene was inserted into pGBKT7 to generate a bait vector. The positive clones were selected on SD/-His/-Leu/-Trp/-Ade/X-α-gal plates by yeast two-hybrid (Y2H) (Chen et al., 2013), and then these clones were identified by sequencing and Blastx. All protocols were carried out in strict accordance with the manufacturer’s instructions.

The protein interactions were further verified using a bimolecular fluorescence complementation (BiFC) assay. GmFBL144 and GmsHSP were fused to nYFP and cYFP, respectively. The fusion constructs were transformed into *Agrobacterium tumefaciens* (GV3101, pSoup-p19) and then co-transferred into epidermal *Nicotiana benthamiana* cell for transient assays (Lu et al., 2009). The fluorescent signals of yellow fluorescent protein (YFP) were observed using a laser confocal microscope (TCS SP8, Leica, Germany).

The full-length *GmCullin1* gene (Glyma.17G025200) was inserted into pGADT7 and pGBKT7. The full-length *GmSkp1* gene was inserted into pGADT7. The full-length *GmFBL144* and *GmSkp1* genes (Glyma.11G079600) were inserted into two distinct multiple cloning sites of the pBridge vector simultaneously. The pairs were co-transformed into Y2H gold yeast competent cells using the PEG/LiAc-mediated method. The interaction between bait and prey proteins were identified on SD/-leu/-Trp and SD/-Leu/-Trp/-His/-Ade/X-α-gal media.

### Determination of Chlorophyll, Malonaldehyde, and Hydrogen Peroxide Content

Chlorophyll content was assessed following previously used methods (Jie et al., 2020). The contents of MDA and H_2_O_2_ were determined though the corresponding test kits (Nanjing Jiancheng Bioengineering Institute, Nanjing, China).

### Statistical Analysis

The results were analyzed using GraphPad Prism 5.0 software. The data are expressed as means ± SD. All experiments were repeated thrice. The statistical significance was considered at the *P* < 0.05 or *P* < 0.01 level, as revealed by *t*-tests.

## Results

### Identification and Bioinformatics Analysis of *GmF-Box* Genes

The *F-box* genes belong to a supergene family, which is a cluster of genes produced by duplication and mutation from an ancestor. Gm19G196400 was defined as an *F-box-like* gene belonging to the *F-box* gene family. To understand Gm19G196400, we performed bioinformatics analysis of the soybean *F-box* gene family. We identified 507 *F-box* genes in the soybean genome database, and we renamed these genes according to the N-terminal domain and chromosomal distribution. Our objective gene, Gm19G196400, is named *GmFBL144* (Supplementary Table 2).

F-box proteins contain other domains at C-terminal besides N-terminal relatively conserved F-box domain. In order to research conveniently, the whole F-box protein family was classified into 11 subfamilies (Supplementary Table 2) and the GmFBL144 belongs to the FBO subfamily (F-box proteins with C-terminal other known domain). The phylogenetic tree analysis showed that most members of the same subfamily tended to cluster in the same evolutionary branch (Supplementary Table 2 and Supplementary Figure 1).

**FIGURE 1:**
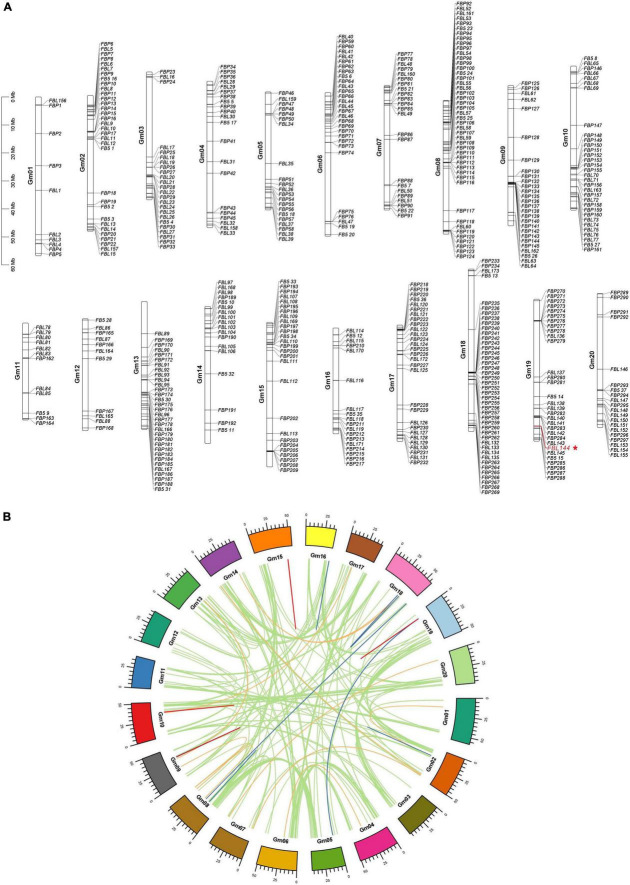
Chromosome distribution and gene duplication analysis of *GmF-box* genes. **(A)** Chromosome distribution of *GmF-box* genes. **(B)** Gene duplication analysis of *GmF-box* genes. The *GmF-box* gene family expansion was primarily caused by WGD/segmental duplication. WGD/segmental duplications gene pairs are shown in green lines; tandem duplication gene pairs are shown in red lines; dispersed duplication gene pairs are shown in orange lines; proximal duplication gene pairs are shown in blue lines.

The distribution of *GmF-box* genes on chromosomes was visualized (Figure 1A). The number of *F-box* genes on chromosome 8 was the largest (48 *F-box* genes), and on chromosome 1 was the lowest (10 *F-box* genes). There was no significant correlation between *F-box* gene number on a chromosome and chromosome length. Gene duplication is the source of evolutionary innovation and main factor in gene family expansion. In this study, gene duplication analysis was performed. 307 WGD/segmental duplication *GmF-box* genes, corresponding to 192 duplicated gene pairs in the entire *GmF-box* gene family, were identified in soybean; 109 dispersed duplication *GmF-box* genes, 44 proximal duplication *GmF-box* genes, and 26 tandem duplication *GmF-box* genes corresponded to 37, 17, and 9 duplicated genes pairs in the *GmF-box* gene family, respectively (Figure 1B). The results of the gene duplication analysis revealed that the *GmF-box* gene family expansion was largely a result of WGD/segmental duplication.

**FIGURE 2:**
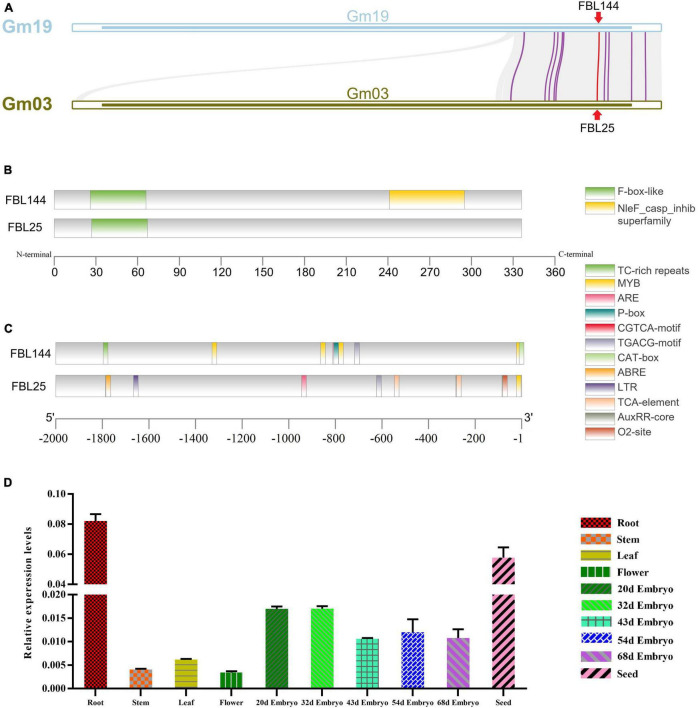
Collinearity analysis and the structural and expression profile analysis of *GmFBL144*. **(A)** Collinearity analysis between chromosome 19 and chromosome 3. **(B)** Gene structure analysis of *GmFBL144* and *GmFBL25*. *GmFBL144* protein shares an F-box domain with GmFBL25, and GmFBL144 has an additional NleF_casp_inhib domain. **(C)** Promoter region analysis of *GmFBL144* and *GmFBL25*. The *GmFBL144* promoter contains four drought response elements (MYB motif) and one stress response element (TC-rich repeats). The *GmFBL25* promoter contains only one drought response element (MYB motif). **(D)** Expression profile analysis of *GmFBL144*. The expression of GmFBL144 in roots, stem, leaves, flowers, embryos of different stages, and seeds. Relative gene expression was calculated using the 2^–Δ^*^Ct^* method. Data represent means ± SE of three biological replicates.

In addition, we analyzed the gene structure and promoter regions of *GmFBL144* and *GmFBL25* that showed the closest genetic relationship with *GmFBL144* (Figures 2A,B and Supplementary Figure 1). The results showed that GmFBL144 had a similar structure to GmFBL25 and adds a NleF-casp-inhib domain (Figure 2B). Furthermore, we found that there are four drought response elements (MYB motif, C/TAACNA/G) and a stress response element (TC-rich repeats, ATTCTCTAAC) in the promoter region of *GmFBL144*, whereas *GmFBL25* only had an MYB motif (Figure 2C). These findings suggest that *GmFBL144* may be involved in drought regulation. We also found that the relative expression of *GmFBL144* was high in roots (Figure 2D). Roots are the main organ of external environment perception. Therefore, we conjecture that *GmFBL144* may be involved in the drought stress response.

### Overexpression of *GmFBL144* Increased Drought Stress Sensitivity in Transgenic *Arabidopsis* Seedlings

**FIGURE 3:**
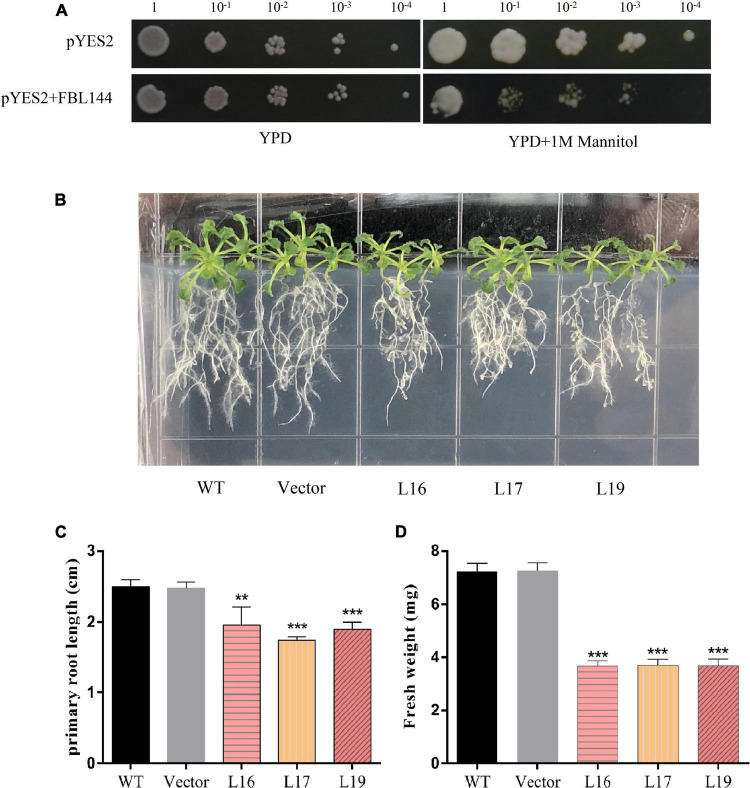
Phenotype of transgenic *Arabidopsis* under simulated drought stress. **(A)** Phenotype of heterologous expression of *GmFBL144* yeast and control under drought stress (1 M mannitol). **(B)** Phenotype of control (wild type and vector) and *GmFBL144*-overexpression lines (L16, L17, and L19) under drought stress (250 mM mannitol). **(C)** Primary root length of control and *GmFBL144*-overexpression lines under drought stress (250 mM mannitol). **(D)** Fresh weight of control and *GmFBL144*-overexpression lines under drought stress (250 mM mannitol). Data represent means ± SE of three biological replicates. Asterisks indicate significant difference applying ANOVA (*P < 0.05; **P < 0.01; ***P < 0.001).

There are multiple drought response elements in the promoter region of *GmFBL144* and our transcriptome data of soybean (Zhou et al., 2020) showed that the *GmFBL144* was down-regulated under drought stress (Supplementary Figure 2). Accordingly, we studied the function of *GmFBL144* in drought stress. In this study, the yeast transient expression system was used. The results showed that the heterologous expression of *GmFBL144* yeast was undergrown compared with the vector control under drought stress conditions (Figure 3A). Next, we carried out the *Arabidopsis* plate drought stress study. Mannitol (250 mM) was used to simulate drought stress in 1/2 MS medium. The results showed that after 10 days of simulated drought stress, the primary root length of *GmFBL144*-overexpression *Arabidopsis* was shorter and the fresh weight was lighter than that of controls (WT and empty vector) (Figures 3B–D).

**FIGURE 4:**
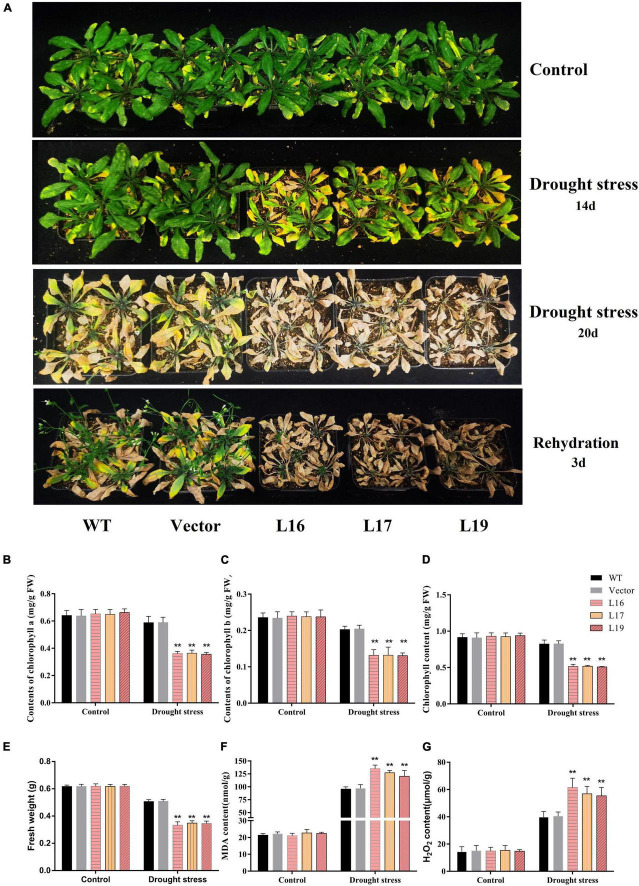
Phenotype and physiological index of transgenic *Arabidopsis* under drought stress. **(A)** Phenotype of control (wild type and vector) and *GmFBL144*-overexpression lines (L16, L17, and L19) under drought stress. **(B–G)** Physiological index of control and *GmFBL144*-overexpression plants under drought stress, including chlorophyll content **(B–D)**, fresh weight **(E)**, MDA content **(F)**, H_2_O_2_ content **(G)**. Data represent means ± SE of three biological replicates. Asterisks indicate significant difference applying ANOVA (*P < 0.05; **P < 0.01; ***P < 0.001).

Similarly, the transgenic *Arabidopsis* showed drought sensitivity under soil drought stress. Under normal conditions, there was no significant difference between controls (WT and empty vector) and the *GmFBL144*-overexpression transgenic lines. After 2 weeks without watering, the foliar loss of *GmFBL144*-overexpression transgenic lines was more serious than that in the controls (WT and empty vector) (Figure 4A). The chlorophyll content of *GmFBL144*-overexpression transgenic lines was lower, and the MDA and H_2_O_2_ content were higher than those of the controls (WT and empty vector) (Figures 4B–G). After 20 days without watering, the *GmFBL144*-overexpression transgenic lines were almost dead; however, the controls (WT and empty vector) were still alive. After 3 days of rehydration, the controls (WT and empty vector) recovered but the *GmFBL144*-overexpression transgenic lines could not (Figure 4A). These results indicate that *GmFBL144* increased plant drought sensitivity through damaging the system of scavenging activated oxygen.

### Subcellular Localization of GmFBL144

**FIGURE 5:**
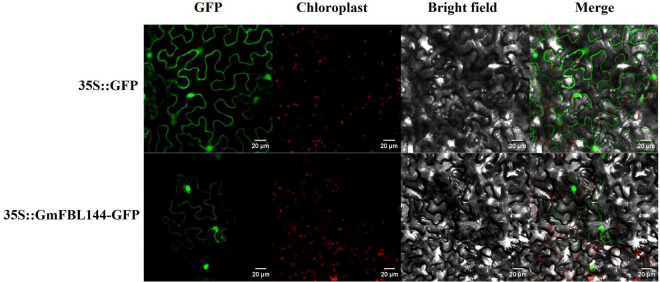
Subcellular localization of GmFBL144 proteins. The fusion constructs and GFP driven by the 35S promoter were transiently expressed in tobacco leaves. Scale bars = 20 μm.

Proteins are the most important biomacromolecules in organisms, and the major performers of life activities. Mature proteins can exert different biological functions in specific subcellular organelles. Thereby, the function of a protein is not only related to its structure but also to its subcellular localization. The subcellular localization prediction websites^4, 5^ forecast that the GmFBL144 protein could be located on the nucleus and chloroplast. At the same time, we investigated the subcellular localization of GmFBL144 using the transient transformation system of tobacco leaves. The fluorescence signal of the fusion protein was mainly located on the nucleus, and a small part also exist on the cell membrane (Figure 5).

### Identification of the Formation Mechanism of the SCF^FBL144^ Complex

**FIGURE 6:**
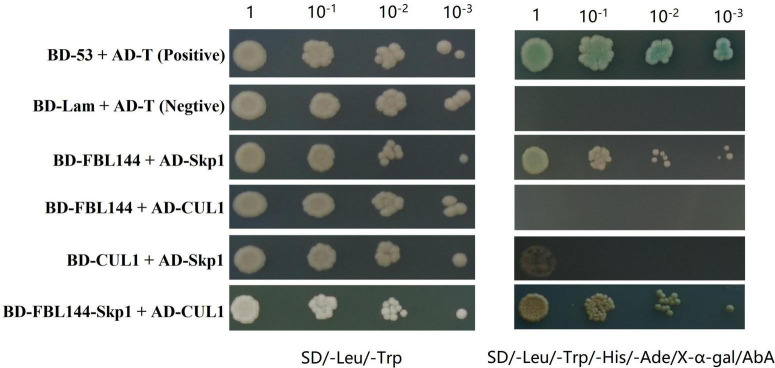
GmFBL144 was a key subunit of SCF*^FBL144^*. GmFBL144 can interact with GmSkp1, and the complex of GmFBL144-GmSkp1 can promote the interaction of GmSkp1 with GmCUL1. The interactions between pGADT7-T and pGBKT7-53, pGADT7-T and pGBKT7-Lam were used as positive and negative control, respectively. AbA, aureobasidin A. X-α-gal, 5-bromo-4-chloro-3-indoxyl-α-D-galactopyranoside.

Studies have shown that most F-box proteins perform functions by forming SCF complexes (Kipreos and Pagano, 2000; Ao et al., 2020). To verify whether GmFBL144 can form SCF complex, we evaluated the interactions among Cullin1 (CUL1), Skp1, and GmFBL144 using Y2H assay. The results showed that GmFBL144 can interact with GmSkp1, cannot interact with GmCUL1; GmCUL1 can interact with GmSkp1 but is weak; the complex of GmFBL144-GmSkp1 can interact with GmCUL1. These results indicated that GmFBL144 is a key subunit of SCF*^FBL144^*, and GmFBL144 can promote the combination of GmSkp1 and GmCUL1 (Figure 6).

### Screening and Identification of GmFBL144 Interacting Proteins

**FIGURE 7:**
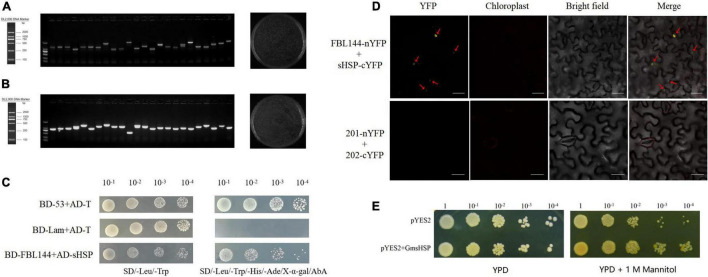
Screening and identification of GmFBL144 interacting proteins. Quality identification of primary library **(A)** and sub-library **(B)**. Recombination rate = successful recombinant clones (24)/total clones (24) × 100%, library capacity = total clones (1300)/spreading volume (0.05 mL) × dilution multiple (100) × total volume (4 mL). Interaction of GmFBL144 with GmsHSP was verification by Y2H **(C)** and BiFC **(D)**. nYFP denotes the YFP N-terminal protein. cYFP denotes the YFP C-terminal protein. Scale bars = 25 μm. **(E)** Phenotype of heterologous expression of *GmsHSP* in yeast under drought stress (1 M mannitol).

In order to further research the mechanism, we screened the interacting proteins through the cDNA library. Because GmFBL144 was primarily located on the nucleus, we constructed a soybean cDNA library of a yeast nuclear system. The primary library capacity was approximately 1.04 × 10^7^, the recombination rate was approximately 100%, and the average insert length was >1,000 bp (Figure 7A). The sub-library capacity was approximately 1.44 × 10^7^, the recombination rate was approximately 100%, and the average insert length was >1,000 bp (Figure 7B). After the soybean cDNA library was constructed, we used BD-GmFBL144 as bait for screening interaction proteins with the Y2H assay. Forty-five positive blue clones were obtained and annotated in Supplementary Table 3. Among these clones, small heat shock protein (sHSP) (GenBank: XP_014626034.1), involved in multiple abiotic stress in some plants (Kuang et al., 2017; Guo et al., 2020), was identified for four times, indicating that it might have the strong interaction with GmFBL144.

The Y2H assay results of the one-to-one interaction verification showed that GmFBL144 can interact with GmsHSP (Figure 7C). We further verified the interaction between GmFBL144 and GmsHSP using BiFC assay. The fusion constructs of FBL144-nYFP and sHSP-cYFP were co-transferred into epidermal *Nicotiana benthamiana* cells for transient assays through *Agrobacterium* infiltration. Yellow fluorescence was observed in cells co-transferred with fusion constructs (Figure 7D), whereas in the negative control, yellow fluorescence was not observed. The results revealed that GmFBL144 can interact with GmsHSP protein.

Small heat shock proteins (sHSPs) are important molecular chaperones, which can prevent damaged protein aggregation caused by stress. sHSP help damaged protein refold to restore biological function by cooperating with other HSPs (HSP100 or HSP70) in the presence of ATP (Lee et al., 2005; Bernfur et al., 2017; Waters and Vierling, 2020). Previous studies have shown sHSP can enhance plant tolerance to external stress (Chauhan et al., 2012; Kuang et al., 2017; Guo et al., 2020). In this study, we identified the function of sHSP through the yeast transient expression system. The results showed that the growth of overexpression of *GmsHSP* yeast was better than that with vector control under drought stress (Figure 7E). This result shows that GmsHSP is a positive regulator of drought stress. The subcellular localization analysis of GmsHSP found that GmsHSP was mainly localized in nucleus and peroxisome (Supplementary Figure 3).

## Discussion

### Gene Duplication

In the process of evolution, soybean has experienced two genome duplication or polyploidization events, resulting in a highly duplicated genome with 75% of genes present in the form of paralogous copies (Shoemaker et al., 1996; Gill et al., 2009; Schmutz et al., 2010). In addition, segmental duplication and tandem duplication are important reasons for increasing gene copies and genetic diversity (Zhao et al., 2018). Previous studies have shown that *F-box* gene family expansion of rice and chickpea mainly caused by tandem duplication (Jain et al., 2007; Gupta et al., 2015). However, in our study, WGD/segmental duplication was the main expansion form of *F-box* gene in soybean. Similarly, the expansion of the *F-box* gene family in wheat, pear, and cotton was dominated by WGD/segmental duplication (Wang G. et al., 2016; Zhang S. et al., 2019; Li et al., 2020a). These differences may be caused by variations in the definition of tandem duplication. Previous studies of the *F-box* gene family have used different definitions on tandem duplication. In contrast, the tandem duplication definition used by MCScanX is more restrictive than that of previous studies (Wang et al., 2012).

The terminal genes of chromosome 19 and chromosome 3 showed collinearity in addition to some *F-box* genes (Figure 2A), which may have contributed to chromosome rearrangement after polyploidization. Similar results have also reported in other studies (Schmutz et al., 2010; Zhang et al., 2018). *GmFBL144* and *GmFBL25* have high homology (Supplementary Figure 1 and Figure 2B), which may be the result of evaluation selection of double-copy gene. Evolutionary selection can lead to loss one of the homologous genes or pseudogenization, and the evolution of new function (Moore and Purugganan, 2003; Semon and Wolfe, 2007; Zhao et al., 2018). Future studies will assess whether GmFBL25 has other functions.

### GmFBL144 Is a Key Subunit of the SCF^FBL144^ Complex

The SCF complex is the main form of the F-box protein that performs function. Some F-box proteins perform function in a non-SCF form in yeast and human (Galan et al., 2001; Nelson et al., 2013), but this has not been found in plants. Interestingly, some F-box proteins can perform functions in both SCF and non-SCF forms in human, for example Fbxo7 (Nelson et al., 2013). Presently, research on plant F-box proteins mainly focuses on their SCF-dependent functions, and there are a large number of F-box proteins in plant. Therefore, it is impossible to rule the existence of the SCF-independent functions of F-box proteins. Previous studies suggested that Cullin1 and Rbx1, Skp1 form a core scaffold (Gagne et al., 2002; Qinxue et al., 2018; Ban and Estelle, 2021). However, in our study, the interaction between Cullin1 and Skp1 was weak, but GmFBL144 can promote the combination of Cullin1 and Skp1 to form SCF*^FBL144^*. This result may be related to the binding of GmFBL144 and GmSkp1, which changes the conformation of GmSkp1 resulting in binding-capacity enhancement of GmCullin1 and GmSkp1.

### GmFBL144 Enhances the Sensitivity of Plants to Drought Stress

**FIGURE 8:**
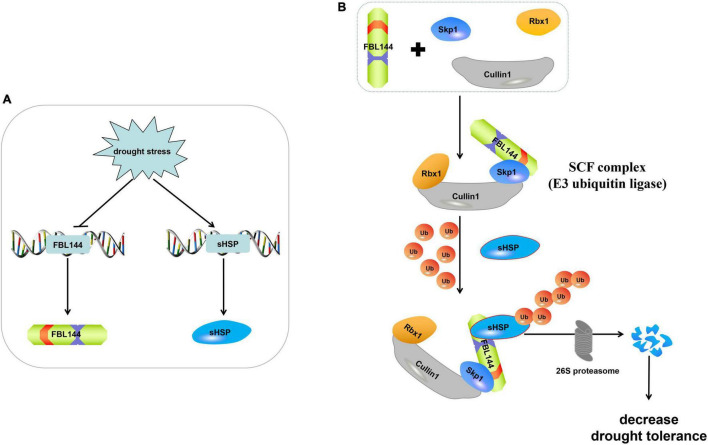
Model for molecular mechanism of GmFBL144. **(A)** In response to drought stress, GmsHSP was increased through the inhibition of *GmFBL144*. **(B)** GmFBL144 can form SCF*^FBL144^* with Skp1 and Cullin1 to promote the ubiquitinated degradation of GmsHSP by 26S proteasome, which reduces plants drought tolerance.

*F-box* genes play an important role in plant growth, development and stress responses. Recently, considerable research has suggested that F-box genes participate in drought stress responses. For example, *Capsicum annuum* Drought-Induced F-box Protein 1 (CaDIF1) is a positive regulator of drought tolerance (Lim et al., 2019). Under drought stress, *F-BOX OF FLOWERING 2* (*FOF2*) positively regulated ABA-induced stomatal closure, resulting in reduced water loss (Qu et al., 2020). In addition, Li et al. (2020b) found that SITLFP8 (Tubby-like F-box protein 8) can enhance tomato drought tolerance by decreasing water loss *via* changing stomatal density. In general, previous studies have shown that *F-box* genes enhance the drought stress tolerance of plants. However, our study showed that *GmFBL144* enhanced the sensitivity of plants to drought stress, potentially caused by the interaction between GmFBL144 and GmsHSP. sHSP as an important molecular chaperone can maintain protein stability (Morris et al., 2010; Papsdorf and Richter, 2014). They also associate with membranes. Sakthivel et al. (2009) found that HspA can stabilize membrane proteins such as the photosystems and phycobilisomes from oxidative damage. Balogi et al. (2008) found that a mutant Hsp17 (Q16R) with increased thylakoid association can improve the tolerance of UV-B damage in synechocystis. In our study, GmsHSP was a positive regulator of drought stress (Figure 7E). When plants were subjected to poor environmental conditions, the homeostasis was maintained through the molecular chaperone and proteolytic systems (Li et al., 2019, 2020d). In our study, *GmFBL144* was down-regulated and *GmsHSP* was up-regulated under drought stress (Supplementary Figure 2). These results indicated that soybean can withstand drought stress by enhancing the chaperone system. Previous studies have found that *F-box* genes positively regulated drought tolerance, likely because F-box proteins maintain intracellular homeostasis *via* the UPS, or increase the content of positive regulatory factors of drought stress. However, in our study, the GmFBL144 may degrade GmsHSP by SCF^FBL144^, destroying the molecular chaperone system and aggravating the protein homeostasis imbalance that leads to drought sensitivity (Figure 8).

## Conclusion

In this study, a total of 507 *GmF-box* genes were identified, and classified into 11 subfamilies. The expansion of the *GmF-box* gene family was primarily caused by WGD/segmental duplication. Under drought stress, the expression of *GmFBL144* was down-regulated (Supplementary Figure 2). Overexpression of *GmFBL144* enhanced sensitivity to drought stress. GmFBL144 can form SCF with Skp1 and Cullin1, and interact with GmsHSP. GmFBL144 may promote sHSP ubiquitination through forming SCF*^FBL144^*, and then the ubiquitinated sHSP is degraded by 26S proteasome (Figure 8B).

## Data Availability Statement

The original contributions presented in this study are included in the article/Supplementary Material, further inquiries can be directed to the corresponding authors.

## Author Contributions

KX and YuZ performed the bioinformatics analysis. YaZ, CF, YiZ, FW, XL, HG, WL, YJ, RS, and XF provided assistance on the experiments. HL and YoZ designed the study and revised the manuscript. All authors reviewed and approved the final manuscript.

## Conflict of Interest

The authors declare that the research was conducted in the absence of any commercial or financial relationships that could be construed as a potential conflict of interest.

## Publisher’s Note

All claims expressed in this article are solely those of the authors and do not necessarily represent those of their affiliated organizations, or those of the publisher, the editors and the reviewers. Any product that may be evaluated in this article, or claim that may be made by its manufacturer, is not guaranteed or endorsed by the publisher.

## Footnotes

1
http://phytozome.jgi.doe.gov

2
http://pfam.xfam.org

3
http://bioinformatics.psb.ugent.be/webtools/plantcare/html/

4
https://www.genscript.com/wolf-psort.html

5
http://www.csbio.sjtu.edu.cn/bioinf/Cell-PLoc-2/
