# Supplementary material for: Soybean F-Box-Like Protein GmFBL144 Interacts With Small Heat Shock Protein and Negatively Regulates Plant Drought Stress Tolerance
Source: Front Plant Sci. 2022 Jun 2;13:823529. doi: 10.3389/fpls.2022.823529 (PMC9201338; doi:10.3389/fpls.2022.823529)
Supplement: Supplementary file 1 [file Data_Sheet_1.doc]

Supplementary Material

# Supplementary Figures

**Supplementary Figure 1.** **Phylogenetic tree analysis of *GmF-box* gene family.** The maximum likelihood phylogenetic tree based on Poisson correction of *GmF-box* genes was constructed with MEGA7. The 507 *GmF-box* genes were classified into 11 subfamilies. FBA subfamily stand for F-box proteins with C-terminal F-box-associated domains; FBD subfamily stand for F-box proteins with C-terminal F-box domains; FBF subfamily stand for F-box proteins with C-terminal DUF domains; FBK subfamily stand for F-box proteins with C-terminal Kelch domains; FBL subfamily stand for F-box proteins with C-terminal leucine-rich repeats (LRR) domains; FBO subfamily stand for F-box proteins with C-terminal other known domains; FBP subfamily stand for F-box proteins with C-terminal phloem protein 2 (PP2) domains; FBR subfamily stand for F-box proteins with C-terminal Arm domains; FBT subfamily stand for F-box proteins with C-terminal with tubby (TUB) domains; FBU subfamily stand for F-box proteins with C-terminal unknown C-terminal domains; and FBW subfamily stand for F-box proteins with C-terminal WD40 domains. *GmFBL144* belongs to the FBO subfamily.

**Supplementary Figure 2. Relative expression of *GmFBL144* and *GmsHSP* under drought stress.** Transcriptomic data for *GmFBL144* and *GmsHSP* responses after 0, 6, and 12 h of drought stress. Under drought stress, *GmFBL144* was downregulated, and *GmsHSP* was up-regulated. Data represent means ± SE of three biological replicates. *P < 0.05; **P < 0.01.

**Supplementary Figure 3. Subcellular localization of GmsHSP proteins.** A.The fusion constructs and GFP driven by the 35S promoter were transiently expressed in tobacco leaves. Scale bars = 20 μm. B. The fusion constructs and GFP driven by the 35S promoter were transformed into Arabidopsis mesophyll protoplasts. The peroxisome marker (SKL) indicated by the red fluorescence were used to co-localize with GmsHSP. Scale bars = 10 μm.

# Supplementary Tables

**Supplementary Table 1.** **List of primer sequences used in qRT-PCR.**

**Supplementary Table 2.** **List of members of the *GmF-box* gene family and renaming of *GmF-box* gene.**

**Supplementary table 3. List of interaction protein of GmFBL144 from the library screening.**
