# Supplementary figures and images for: Soybean F-Box-Like Protein GmFBL144 Interacts With Small Heat Shock Protein and Negatively Regulates Plant Drought Stress Tolerance
Source: Front Plant Sci. 2022 Jun 2;13:823529. doi: 10.3389/fpls.2022.823529 (PMC9201338; doi:10.3389/fpls.2022.823529)

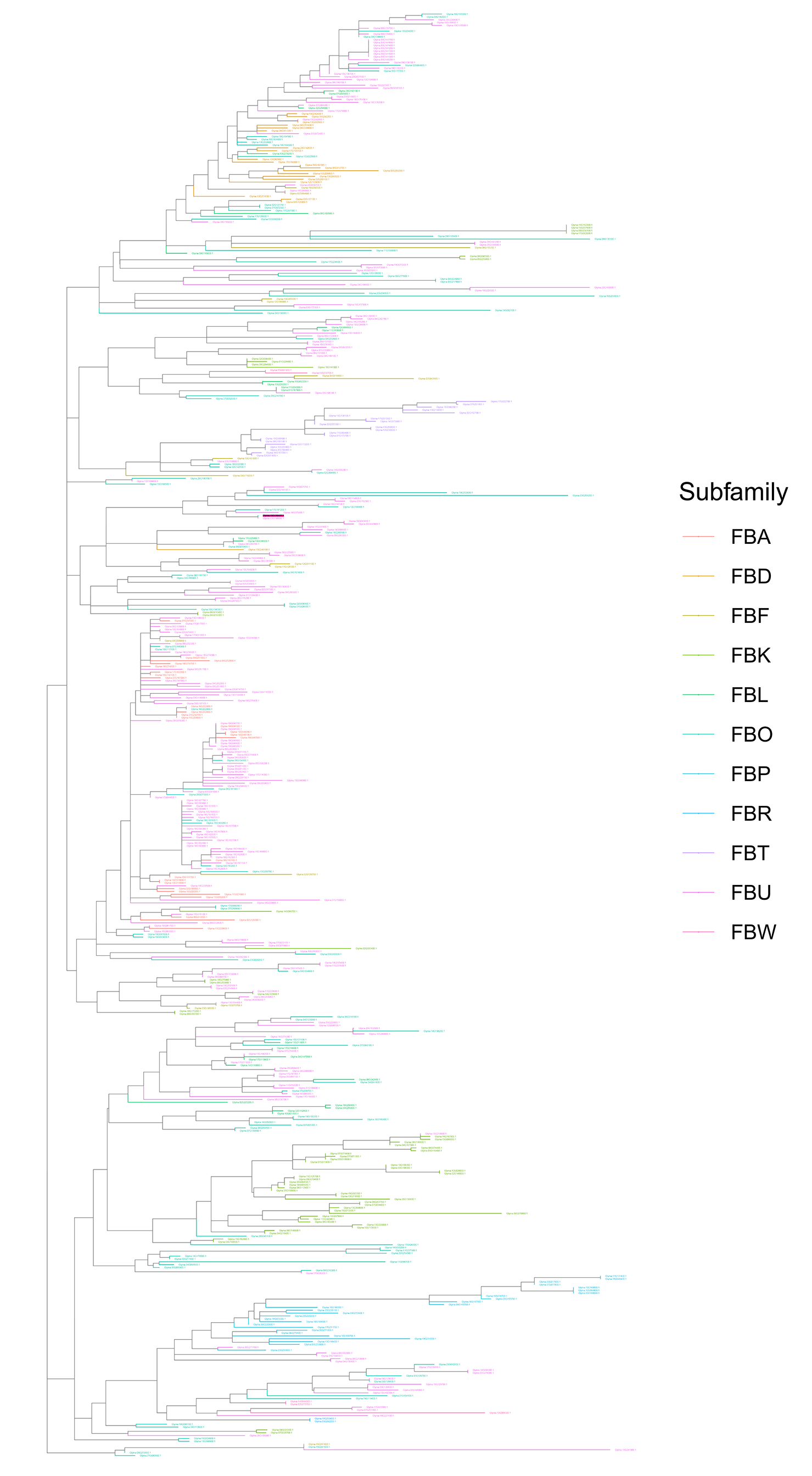

Supplement: Supplementary file 6 [file Image_1.TIF]

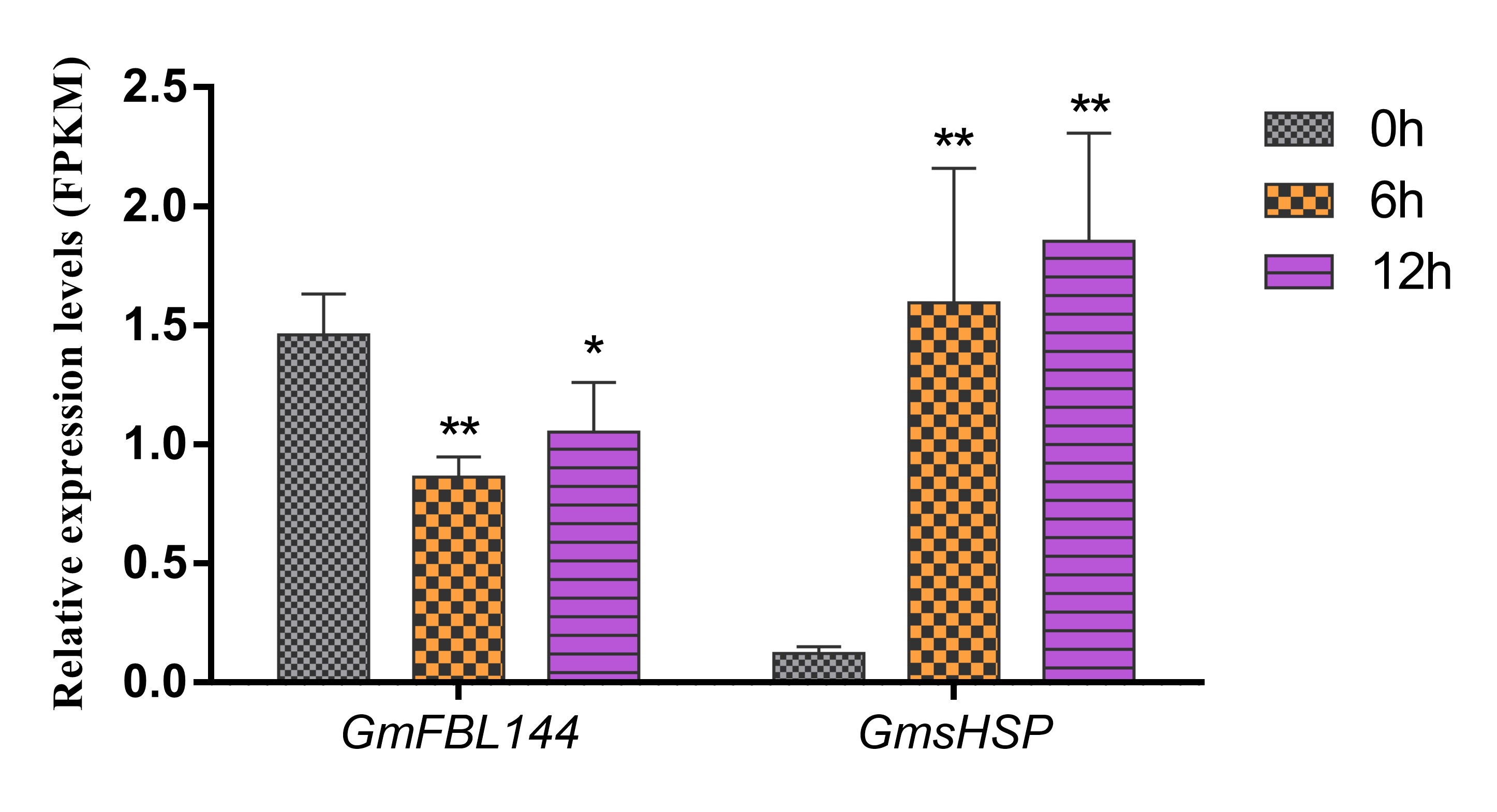

Supplement: Supplementary file 7 [file Image_2.JPEG]

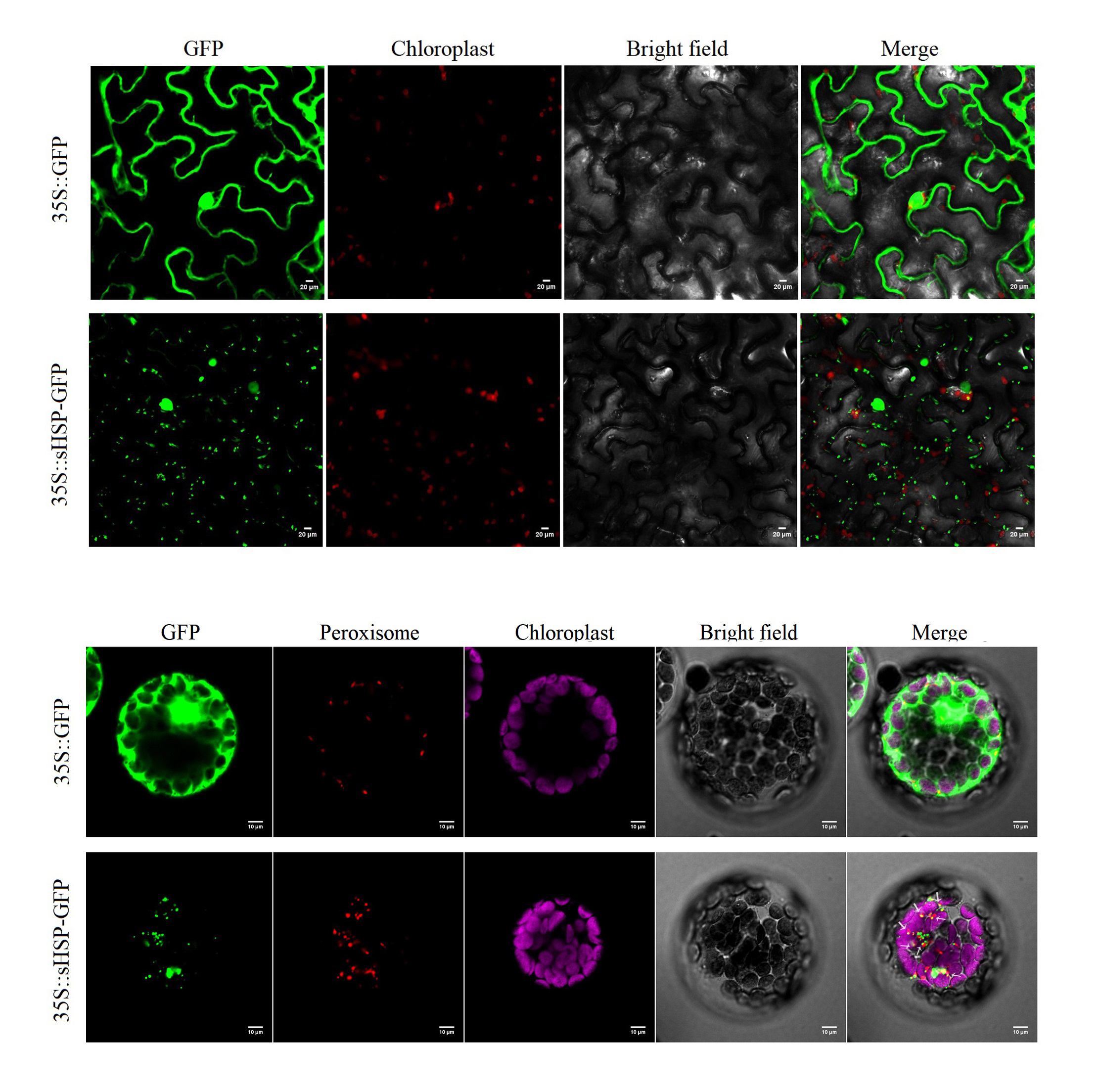

Supplement: Supplementary file 8 [file Image_3.JPEG]
